# Acupuncture for postoperative gastroparesis syndrome: A protocol for systematic review and meta-analysis

**DOI:** 10.1097/MD.0000000000032468

**Published:** 2022-12-23

**Authors:** Wei Li, Ning Zhang, Mengmeng Xiao, Li Liu, Lan Yao

**Affiliations:** a Department of Pain, Peking University International Hospital, Beijing, China; b Department of Anesthesiology, Peking University International Hospital, Beijing, China; c Department of Retroperitoneal Tumor Surgery, Peking University International Hospital, Beijing, China; d Department of Pain, Peking University Third Hospital, Beijing, China; e Department of Pain and Department of Anesthesiology, Peking University International Hospital, Beijing, China.

**Keywords:** acupuncture, meta-analysis, postsurgical gastroparesis syndrome, protocol

## Abstract

Postsurgical gastroparesis syndrome (PGS) is an early common complication after upper abdominal surgery, especially which occurs mainly after gastroduodenal operation. Acupuncture, a complementary and alternative therapy, has been widely used in China because of its significant effect and few side effects. We conduct a protocol for systematic review and meta-analysis to assess the efficacy and safety of acupuncture for the treatment of PGS.

**Methods:**

This systematic review was registered in the PROSPERO network (registration number: CRD42022369167). We will follow the Preferred Reporting Items for Systematic Reviews and Meta-analysis Protocol to accomplish the study. Following databases will be searched: PubMed, MEDLINE, EMBASE, Cochrane Library, China National Knowledge Infrastructure, Wanfang data, Chinese Scientific Journals Database, and China biomedical literature database. All randomized controlled trials (RCTs) on the application of acupuncture in the treatment of patients with PGS will be included. The risk of bias of the included studies will be assessed using the Cochrane tool of risk of bias. All statistical analyses will be conducted using the STATA13.0 software.

**Results:**

This study is ongoing and the results will be submitted to a peer-reviewed journal for publication.

**Conclusion:**

The conclusion of this review will provide evidence to judge whether acupuncture is an effective intervention for patient with PGS.

## 1. Introduction

Postsurgical gastroparesis syndrome (PGS) is a complex disorder characterized by post-prandial nausea and vomiting, and gastric atony in the absence of mechanical gastric outlet obstruction, and is often caused by operation at the upper abdomen, especially by gastric or pancreatic resection, and sometimes also by operation at the lower abdomen, such as gynecological or obstetrical procedures.^[1,2]^ PGS after gastrectomy has been reported in many previous studies, with an incidence of approximately 0.4% to 5.0%.^[3,4]^

Patients frequently present with marked weight loss and malnutrition requiring hospitalization and prolonged parenteral nutrition. These symptoms can be disabling and are frequently unresponsive to drug therapy. Gastric reoperations are frequent and usually unsuccessful. The cause of PGS has not been identified and its mechanism is indefinite.

Traditional Chinese medicine, which includes acupuncture and moxibustion, and Chinese herbal products, has been integrated as an important part of healthcare and has been used to treat various diseases.^[5,6]^ Acupuncture, a complementary and alternative therapy, has been used in China for thousands of years and has become increasingly popular in western countries because of its significant effect and few side effects.^[7,8]^ In particular, since acupuncture is a non-pharmacological intervention, it has the advantage of being free from herb-drug interactions with a high potential for use as an adjuvant therapy along with conventional medications. Both basic research and clinical practice have proved that acupuncture has an obvious effect on PGS,^[9]^ but the therapeutic mechanism has not been very clear yet. Perhaps it may modulate gastric motility through the activation of sympathetic efferent nerve fibers or vagal nerve fibers.

Though PGS is not a life-threatening disease, patients often suffer from a poor quality of life, which is regarded as an economic burden on society. Currently, evidence for the clinical use of acupuncture in PGS is still inconclusive. Therefore, we conduct a protocol for systematic review and meta-analysis to assess the efficacy and safety of acupuncture for the treatment of PGS.

## 2. Methods

This systematic review was registered in the PROSPERO network (registration number: CRD42022369167). We will follow the Preferred Reporting Items for Systematic Reviews and Meta-analysis Protocol^[10]^ to accomplish the systematic review protocol. This study is conducted for the secondary collection and analysis of original data; therefore, ethical approval is not required.

### 2.1. Inclusion criteria

#### 2.1..1. Type of study.

All randomized controlled trials (RCTs) on the application of acupuncture in the treatment of patients with PGS will be included with no language limitation. However, animal studies, case reports, case series, commentaries, reviews, non-controlled trials, and other studies that are repeatedly published will be excluded.

#### 2.1..2. Types of participants.

Patients who are diagnosed with PGS will be included, without limits on gender, race, nationality, and medical units.

#### 2.1..3. Types of interventions and comparisons.

Interventions can be any type of acupuncture; multiple control interventions will be included: no treatment, placebo, and other interventions (e.g., cupping therapy, drugs, physical interventions, moxibustion). If the interventions and comparisons both contain acupuncture, the study will be excluded. Interventions of acupuncture combined with other therapies will be included only if these combinations are compared to other therapies.

#### 2.1..4. Types of outcome measures.

The primary outcome is the effective rate, which is categorized as cure, markedly effective, effective, or ineffective according to clinical symptoms; the secondary outcomes include quality of life and adverse effects.

### 2.2. Search methods

Following databases will be searched: PubMed, MEDLINE, EMBASE, Cochrane Library, China National Knowledge Infrastructure, Wanfang data, Chinese Scientific Journals Database, and China biomedical literature database. We will select the eligible studies published up to December, 2022. The search uses a combination of subject words and free words, and the search strategy is determined after multiple researches. The search terms include acupuncture, gastroparesis syndrome and randomized. Meanwhile, we will search the literature included in the research reference and original literature which are subject-related and included by systematic reviews. The search strategy of PubMed will be shown in Table 1.

**Table 1:** Search strategy used in PubMed database.

Number search terms
#1 postoperative gastroparesis syndrome [Ti/Ab]
#2 postsurgical gastroparesis syndrome [Ti/Ab]
#3 gastric paralysis syndrome [Ti/Ab]
#4 delayed gastric emptying [Ti/Ab]
#5 gastric emptying disorder [Ti/Ab]
#6 gastroparesia [Ti/Ab]
#7 gastroplegia [Ti/Ab]
#8 paralysis of stomach [Ti/Ab]
#9 #1 OR #2 OR #3 OR #4 OR #5 OR #6 OR #7 OR #8
#10 acupuncture [Ti/Ab]
#11 traditional manual acupuncture [Ti/Ab]
#12 acupoint [Ti/Ab]
#13 dermal needle [Ti/Ab]
#14 #10 OR #11 OR #12 OR #13
#15 randomized [Ti/Ab]
#16 randomly [Ti/Ab]
#17 blind [Ti/Ab]
#18 #15 OR #16 OR #17
#19 #9 AND #14 AND #18

### 2.3. Study selection

**Figure 1.**
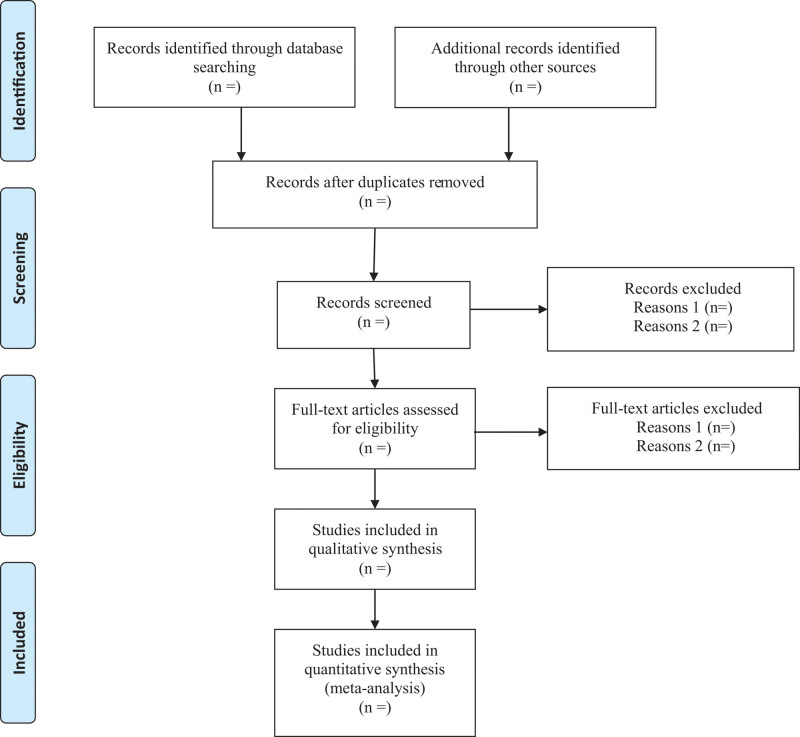
Flow diagram of study selection process.

Researchers will discuss and determine the screening criteria within the group before searching the studies. The corresponding research members will import the retrieved studies into the document management system of EndnoteX7 for repetition removal. We will then exclude the apparently unqualified literature by reading the headings and abstracts, and determine the final included literature by reading the full text, discussing within the group and contacting the author to know more about the research details. The final list of included studies will be converted to the format of Microsoft Excel. Both the information retrieval and the literature screening will be independently operated by 2 research members. Finally, another research member will resolve the inconsistency and check the final included studies. Study selection is summarized in a PRISMA flow diagram (Fig. 1).

### 2.4. Data extraction

Data extraction will be conducted by 2 researchers using EpiData 3.1 software for double entry. Data for collection include age, sample size, disease diagnosis, combined disease, interventions and details about the control group, follow-up, outcomes, and adverse event. Any disagreement on data collection will be resolved through discussions or negotiations with the third arbitrator. If the data provided in the study are unclear, missing, or presented in a form that is not extractable or difficult to extract reliably, we will contact the author of the study for clarification.

### 2.5. Risk of bias assessment

The risk of bias will be assessed independently by 2 authors using the Cochrane tool of risk of bias (V.5.1.0).^[11]^ The following items will be assessed: random sequence generation (selection bias), allocation concealment (selection bias), blinding (performance bias and detection bias), incomplete outcome data (attrition bias), selective outcome reporting (reporting bias), and other bias. The judgments of evaluated domains will include high, low, and unclear. Disagreements will be resolved by discussion by arbiter.

### 2.6. Statistical analysis

Two researchers respectively enter the data into the STATA13.0 software. Mean differences with a 95% confidence interval (CI) are calculated to assess the effect size for continuous outcome data. Risk ratio with a 95% CI are used as effect size for dichotomous data. Inverse variance method and Mantel-Haenszel analysis method are used for continuous variables and dichotomous variables, respectively.^[12]^ The heterogeneity among the trials is assessed for significance with Q and quantified with I^2^.^[13]^ Statistically significant is set at the *P* value < .10. If the studies are homogeneous or the statistical heterogeneity is low, we use the fixed effect-model. While, random-effects model is applied when the statistical heterogeneity is moderate or high.

### 2.7. Sensitivity analysis

Sensitivity analysis will be used to test the quality of the research contained in the sampled documents. The stability of the conclusions can be tested by re-analyzing the conclusions by inputting missing data and changing the type of research.

## 3. Discussion

PGS is an early common complication after upper abdominal surgery, especially which occurs mainly after gastroduodenal operation, and also occurs after non gastroduodenal operation.^[14,15]^ The pathogenesis of PGS is not completely understood, which is considered to be related to many factors, such as operative procedure, mental factor, neural factors anesthesia factor, underlying diseases, changes of diet structure, and so on.^[16]^

Acupuncture is a widely used non-pharmacological intervention in complementary medicine that classically involves the insertion of fine needles into certain locations that are considered emerging points of vital energy (the “qi” of traditional Chinese medicine).^[17,18]^ This study may provide evidence for the use of acupuncture in treating PGS. Besides its efficacy, adverse events of acupuncture should be more thoroughly investigated and transparently reported to improve the current lack of evidence for the safety of acupuncture in this condition. In addition, the optimal mode, frequency, and duration of acupuncture should be investigated to achieve the best patient-relevant outcomes.

## Author contribution

**Conceptualization:** Ning Zhang.

**Data curation:** Mengmeng Xiao.

**Investigation:** Li Liu.

**Writing – original draft:** Wei Li.

**Writing – review & editing:** Lan Yao.
